# The Risk Factors for the Wearing-Off Phenomenon in Parkinson's Disease

**DOI:** 10.7759/cureus.10729

**Published:** 2020-09-30

**Authors:** Kunal Raja, Sonam Ramrakhia, Kapeel Dev, Wajeeha Shahid, Hamza Sohail, Muhammad Khizar Memon, Sidra Memon

**Affiliations:** 1 Internal Medicine, Shaheed Mohtarma Benazir Bhutto Medical University, Larkana, PAK; 2 Medicine, Liaquat University of Medical and Health Sciences, Sukkur, PAK; 3 Medicine, Mustafai Trust Central Hospital, Sukkur, PAK; 4 Internal Medicine, Ghulam Muhammad Mahar Medical College, Sukkur, PAK; 5 Internal Medicine, Jinnah Sindh Medical University, Karachi, PAK; 6 Neurology, Jinnah Sindh Medical University, Karachi, PAK; 7 Internal Medicine, Liaquat University of Medical and Health Sciences, Sukkur, PAK

**Keywords:** parkinson' s disease, wearing off, pakistan

## Abstract

Introduction: First-line treatment of Parkinson's disease (PD) includes a dopamine analog, levodopa, administered in combination with carbidopa to increase efficacy. Wearing-off (WO) phenomenon is a frequent complication which is defined as a reoccurrence of motor and non-motor symptoms during levodopa free interval, which has a negative impact on the quality of life of patients. Through this study, we aim to determine risk factors that lead to the manifestation of the WO phenomenon among patients presenting in our out-patient department of a tertiary care hospital in Pakistan.

Method: A observational case-control study was conducted from April 2019 to December 2019 in a tertiary care hospital in Pakistan. A total of 101 patients who had PD were included in the study. They were randomized into two groups i.e. patients who had WO phenomenon (59 participants) and patients who did not experience WO (42 participants) phenomena. Patients were evaluated based on a self-administrated questionnaire. A p-value of less than 0.05 was considered significant.

Result: WO was significantly higher in those patients who had earlier onset of Parkinson (59 ± 10 vs. 65 ±8; p<0.002) and had the disease for a longer duration (7.9±5.1 vs. 5.6±3.1, p<0.002). Other findings included, there was more risk of WO in patients on anti-parkinsonian treatment for longer duration (7.2±5.1 vs. 3.9±3.5, p<0.010) and on longer duration on levodopa treatment (6.9±4.9 vs. 3.1±2.8, p<0.0001).

Conclusion: Our study demonstrated several factors which are responsible for the WO phenomenon. This will aid neurologists to consider these risk factors while prescribing different treatment modalities for the disease to improve efficacy and mitigate WO effect among patients, specifically while advising levodopa.

## Introduction

Parkinson's disease (PD) is a slowly progressive neurodegenerative movement disorder that predominantly affects the dopamine-producing (dopaminergic) neurons in the substantia nigra, the area of the brain that functions to modulate movement and reward systems, resulting in tremors, bradykinesia, limb rigidity, and gait and balance problems [1]. Affecting 1-2 people per 1000 of the population at any time, the prevalence of PD increases with age with more than 1% of the population above 60 years affected [2]. Several environmental and genetic factors have been identified that lead to the development of PD, but in most cases, the cause is idiopathic [2].

Mostly diagnosed on the bases of its cardinal signs and symptoms identified on history and examination, PD is mainly managed symptomatically using both pharmacological and nonpharmacological methods [3]. Levodopa, a synthetic analog of dopamine, is the first-line treatment of PD, mostly given with carbidopa (decarboxylase inhibitor) to prevent the peripheral breakdown of levodopa before it reaches the brain [3,4]. Defined as a recurrence of motor and non-motor symptoms during the levodopa free interval, WO significantly decreases the quality of life of patients with PD, making it necessary to identify risk factors that favor the occurrence of WO in these patients [4]. Wearing-off (WO) phenomenon is a frequent complication encountered with the treatment of levodopa, the incidence of which gradually increases to affect almost all PD patients 10 years after the initiation of the drug [5,6]. Globally, various studies have been conducted to identify these risk factors which include younger age at presentation of symptoms, female sex, long duration of treatment with levodopa, higher doses of levodopa and the use of dopamine agonists, catechol-O-methyl transferase (COMT) inhibitors, and other anti-parkinsonian medications [4,7-8]. To know the incidence and identify these risk factors for WO in PD patients in Pakistan, this study was conducted.

## Materials and methods

This observational, cross-sectional study was conducted from April 2019 to Dec 2019 in the Neurology department in a tertiary care hospital, Pakistan. One hundred and one patients with PD were enrolled after informed consent. The participants were divided into two groups: patients with WO and patients without WO. Patients demographics including gender, current age, age at the time of diagnosis of PD, disease duration, anti-Parkinson treatment duration, levodopa treatment duration, and daily levodopa dose was noted in a self-structure questionnaire.

Analysis was done using the Statistical Package for Social Science (SPSS) software, version 23.0 (SPSS Inc., Chicago, IL, USA). Numerical data was presented as means ± standard deviations (SD). Categorical data were presented as frequencies and percentages. Chi-square and independent t-test (unpaired t-test) was applied as appropriate. P value of less than 0.05 indicated that null hypothesis was not valid and there was a difference between variables. 

## Results

Out of 101 participants, there were 59 (58.4%) patients who exhibited the symptoms of WO and 42 (41.6%) who did not. WO was significantly higher in those patients who had earlier onset of Parkinson (59 ± 10 vs. 65 ±8; p<0.002), had the disease for a longer duration (7.9±5.1 vs. 5.6±3.1, p<0.002), on anti-parkinsonian treatment for longer duration (7.2±5.1 vs. 3.9±3.5, p<0.010), and on longer duration on levodopa treatment (6.9±4.9 vs. 3.1±2.8, p<0.0001) (Table 1).

**Table 1:** Comparison of Risk Factors for Case and Control Group ^a^ means Dependent t-test applied between case (wearing off) and control (not wearing off) * means non-significant result ** means significant result

Characteristics	Overall (101)	Case Group Wearing-off (59)	Control Group Non-wearing-off (42)	P value ^a^
Male : female	46 : 55	29: 30	17: 25	p=0.74*
Age (y)	66 ± 8	64 ± 9	69 ± 7	p< 0.003**
Age of onset (y)	61. ± 10	59 ± 10	65 ±9	p<0.002**
Disease duration (y)	7.2±4.4	7.9±5.1	5.6±3.1	p<0.010**
Medication				
Anti-parkinsonian treatment duration (y)	5.7±4.0	7.2±5.1	3.9±1.8	p<0.0001**
L-dopa treatment duration (y)	5.1±3.8	6.9±4.9	3.1±2.8	p<0.0001**
Daily L-dopa dosage (mg)	480.2 ±201	560 ± 273	320 ±118	p<0.0001**
Dopamine agonist (n)	62 (61.3%)	41 (69.4%)	21 (50%)	p=0.04**
Monoamine oxidase-B (MAO-B) inhibitor (n)	32 (31.6%)	17 (28.8%)	15 (35.7%)	p=0.53*

## Discussion

In this study, young age of Parkinson onset, longer duration of disease, longer duration on levodopa treatment, higher dose of levodopa, and dopamine agonist was associated with WO phenomena. Several studies have reported younger-onset PD as a risk factor for the development of WO phenomenon irrespective of the epidemiology. A Stalevo Reduction in Dyskinesia Evaluation in Parkinson's Disease (STRIDE-PD) study was conducted to identify several predictive factors for WO, among which younger-onset PD was the strongest factor [7]. Studies from other Western and Asian countries mainly Japan and China, have also reported a low incidence of WO and dyskinesia in patients with older-onset PD than in those with younger-onset PD [4,7-11]. Another study found the incidence of dyskinesia, WO and on-off phenomenon to be higher in younger-onset PD (age of onset 21-50 years) patients after comparing 22 autopsy-proven early-onset PD patients with 44 autopsy-proven late-onset PD patients (age of onset ≥ 65 years) [12]. In this present study, the mean age of onset of PD patients with WO phenomenon was lower (59.3 years) when compared to PD patients without WO phenomenon (64.9 years), confirming that young age at the onset of PD is a risk factor for the development of WO.

The present study showed there was an equal risk for males and females for developing WO phenomena. This was contrary to the previously conducted Japanese study, which demonstrated the odds ratio (OR) for WO in female PD patients to be 6.49 in comparison to males [4]. A STRIDE-PD study, by a multivariate analysis, also reported the incidence of WO to be much higher in female PD patients [7]. This can be partly explained by the increased dosage (per kg) of levodopa required in female patients due to lower body weight than males, environmental factors, and the influence of oestrogen and uric acid (UA) [8,13-14].

In this study, Parkinson patients on levodopa were at greater risk of developing WO. The present study also demonstrated that the longer the treatment of PD patients with antiparkinsonian medication including Levodopa, the higher the chances of developing WO symptoms. In their study to identify risk factors and safe dosage of levodopa for WO phenomenon in Chinese PD patients, Chen et al. concluded that the high levodopa dose was associated with WO [8]. This is supported by the Earlier versus Later levodopa Therapy in Parkinson's disease (ELLDOPA) and STRIDE-PD study that found that WO is directly related with the increasing dose of levodopa, with patients developing symptoms of WO within 40 weeks of receiving 600 mg of levodopa [7,15]. The present study also identifies the increased dosage of levodopa as a risk factor for WO in PD patients.

To the best of our knowledge, this is the first study in a local setting that studied the risk factors associated with WO phenomena in Parkinson patients. Further large-scale trial is needed to identify the risk factors associated with WO in Parkinson patients and help manage the symptoms so there is no hindrance in the management of PD.

## Conclusions

In conclusion, younger age at the disease onset, levodopa treatment, and high daily dosage of levodopa were identified as the risk factors for the WO phenomenon in Pakistani PD patients. Careful prescribing of levodopa and other antiparkinsonian drugs and knowledge of these common risk factors for WO must be necessary for all physicians to reduce the incidence of WO.
